# The Psychologic Side of Anesthesia

**Published:** 1913-03

**Authors:** Harry R. Trick

**Affiliations:** Buffalo


### The Psychologic Side of Anesthesia

By DR. HARRY R. TRICK
Buffalo

THE introduction of the modern inhalation anesthetics brought with it a problem that has not yet been solved. This problem is how to obtain the desired effects of anesthetics and eliminate the lethal effects—in other words, what can we do to insure the safety of the patient?

Many attempts have been made to modify the toxicity of these agents by combining them in various ways, but the resulting mixture has never proved to be any more satisfactory than its several elements used alone or in sequence, so these mixtures have never attained a permanent popularity.

Also, a great variety of mechanical devices have been produced to deliver the drug in measured amounts and in pleasanter form, but the machine does not prevent over-administration.

Since we cannot change the properties of these drugs, not devise machines that will relieve us of responsibility during their administration, we must look elsewhere for the solution of the problem.

The administration of an anesthetic implies either that there is need for some surgical procedure or that there is some condition requiring the use of a drug of great potency for its relief. The dangers incident to or a part of such procedure or illness will always beset the patient, and deaths from such causes should not be charged to the anesthetic.

In the past, our statistics of anesthetics have not clearly differentiated between deaths due to an anesthetic and deaths during the administration of an anesthetic. The difference is obvious and no fair deduction can be drawn from them until some standard of what constitutes a death from an anesthetic has been established.

After a recent study of these statistics, covering the Crimean war and including all climates, Gwathmey reports that the inhalation anesthetics do not differ so much in their lethal qualities as we have been taught to believe, and that "no anesthetic death is possible today except from experimenting on new lines, or from carelessness or ignorance."

Professor Henderson of Yale says that the more he studies the situation the more convinced he becomes that deaths under anesthesia are not due to anesthetics but to anesthetists.

A little thought will show how nearly true these statements are, and suggests the need for a study of the individuals concerned in the production of the anesthetic state. Although the methods of administration have been much improved, we still hear too much about the shock of the anesthetic.

It matters little which particular theory of shock we accept, whether it be the nerve exhustion theory of Crile, the respiratory or acapnia theory of Henderson, the inhibition theory of Meltzer, or the cardiac and vascular theory of Howell and Bloodgood, the causes remain the same—namely, pain, hemorrhage, the toxic properties of anesthetics, exhaustion, fear and excitement.

In elective or deliberate surgery the first four causes are being successfully combated by careful handling of tissues, control of hemorrhage, minimal amounts of anesthetics and preliminary rest, but fear and excitement still remain.

Since fear and excitement are psychic disturbances, they should be treated by psycho-therapy. The use of morphine as a preliminary measure to deaden the perceptive centres has been very widely adopted but is open to some criticism as a routine practice; although it is said that even Bernheim does not hesitate to use it in intractable cases.

Morphine, moreover, comes relatively late in the pre-operative treatment of a case and should be used as a narcotic only when suggestion fails.

Fear and excitement begin their destructive processes with the first intimation of the need of an operation, and *then* is the time the psychologic side of the anesthetic should be begun, and every physician can adapt himself to the case at hand if he desires so to do. Any physician who simply tells a patient that he needs a certain operation fails in his duty. It is quite as important to picture an attractive outlook and to remind him of the blessings of the modern anesthetics.

The beneficial effect of suggestion is particularly striking in children. Only recently the writer had occasion to reduce a fracture of the radius in a ten-year-old boy whose reputation at home was that he was a "cry-baby." You will recognize the circumstances and the unattractive outlook. By actual count only 20 drops of chloroform were used to produce anesthesia, with no period of excitement and no subsequent vomiting. No morphine was used. This case is cited because it is so ordinary; it has no unusual features. Anyone can secure a similar result who is willing to take the time and will go into it enthusiastically; but he must be enthusiastic. In cases like the one just cited this method is an actual time-saver as well as a possible life-saver. In order to avoid frightening very young children they may be anesthetized while asleep, or while being held in the mother's arms.

During the first half of the nineteenth century a great many surgical procedures were successfuluy carried out with no other aid than suggestion, although it was called by another name, with no pain, and, strangest of all, with no shock. Dr. Eliotson in 1843 reported numerous cases of surgical operations without pain.

Bramwell reports a public demonstration of this method of producing anesthesia where the post-operative condition of the patients was so pleasant that in the words of the reporter "if it had not been for the bandages and splints one might have thought they were a picnic party."

Esdaile, while serving with the British Army in India, removed hundreds of the huge scrotal tumors peculiar to that part of the world, without pain and without shock. He performed many other major operations of his day, such as breast amputations, with uniform success, and others have done equally as well even in our own day. The literature of psycho-therapy is filled with such reports and affords interesting and inspiring reading.

It is generally conceded that in these various painless and shockless operations the anesthesia obtained was really the work of suggestion, no matter whether produced by passes, magnets, tractors, commands or mirrors. It all goes to prove that "man is a suggestible animal," and in the pre-anesthetic state he is peculiarly open to suggestion. His fear prompts him to grasp at every straw which might tend to increase his safety; therefore, he heeds our suggestions eagerly.

Suggestion does not consist entirely of verbal instruction, although it depends largely upon it. We seldom appreciate the importance of our deportment. The patient's fear is enhanced or allayed by the construction he puts on our actions and manner of speaking; so it behooves us to so act and speak that we inspire our patient with optimism. A patient's fear is seldom of the operation but often of the anesthetic and is largely due to his lack of information or imperfect information. This can be overcome to a great extent by proper instruction as to the nature and mode of action and method of administration of the particular anesthetic to be used, but bearing particularly upon the fact that a large part of the success depends upon the patient himself; showing him how non-resistance means a lessened amount of anesthetic and a pleasanter post-operative experience. This last statement makes a profound impression upon the minds of most patients and is frequently the only point to call to their especial attention. Be sure not to say too much. Over-solicitious concern may develop fear even in those who have no preformed ideas. If men in the past have succeeded in producing anesthesia by suggestion, we certainly ought to be able to secure aquiescence to an anesthetic by its aid. Occasionally we find a case of unreasoning fear, when morphine is clearly needed.

Suggestion as used in this connection means instruction, advice, encouragement, and all other means for securing the faith of the patient. When the time arrives for the administration of the anesthetic the patient should again be reminded of his part; tell him something about the various sensations he will experience and that they are a perfectly natural thing to expect, but repeatedly advising him to go to sleep. A few drops of a 25 per cent. solution of the essence of orange on the mask, as suggested by Gwathmey and Woolsey, will overcome the odor of ether and thus favor early induction by ether.

The surroundings should be conducive to sleep; the anesthetic should be given in a quiet, darkened room; there should be no slamming of doors, and there should be no talking except by the anesthetist, in a soothing tone. If these few directions be followed the patient becomes practically *en rapport* with the anesthetist, and in most instances there will be no period of excitement.

But the benefits of suggestion do not end here. A responsive patient actually requires less anesthetic, suffers practically no shock and rarely vomits. The convalescence is much pleasanter and shorter than it otherwise would be. Local anesthetics are much more effective when combined with suggestion.

Boris Sidis, in his Psychology of Suggestion advances a theory that a patient in a state of "increased suggestibility" tends to prevent the transmission of nocuous impulses by retraction of the neurons, with consequent loss of continuity of the nerve elements. No matter what the change in the nerve elements under suggestion may be it is more desirable than Crile's anoci-association, which it strongly suggests since the function is not inhibited by poisonous drugs. At any rate the removal of fear has been shown both clinically and experimentally to increase the patient's resistance to other shock-producing factors.

The good results reported in connection with the use of the various machines would very probably have been secured by the men who operate them if they had worked with the simplest form of administration, because their patients had faith in them and they had faith in themselves. In the last analysis an ideal anesthetization depends upon the anesthetist.

The use of suggestion as an anesthetic is not a practicable procedure in our modern operating-rooms, but there is no good reason why it should not be used in conjunction with anesthetics, thereby rendering small amounts more effective and avoiding the danger of over-administration; and that is the only point in this paper, and the only plea is for its more extensive application.

### Bibliography.

Psychology Applied to Medicine........................Wells
The Psychologic Side of Medicine (Reprint)..........Barker
Mental Therapeutics...............................Hudson
The Psychic Treatment of Nervous Disorders..........Dubois
Suggestive Therapeutics............................Bernheim
Hypnotism and Treatment by Suggestion............Bramwell
The Psychology of Suggestion.........................Sidis
Phycho-therapy ......................................Walsh
Phylogenetic Association...............................Crile
Anoci-Association .....................................Crile
Suggestion as an Aid to Anesthetics.................Munro

---

The Optometry Question. Louis Striker (abstracted from *Ophthalmology* of Oct., 1912, in *Jour. Oph. and Oto-Laryng.*) says that a united profession is necessary if we are to prevail in this fight. The general profession ought never to refer a case of refraction to an optician, a not uncommon practice today and on which opticians pride themselves. He is sure that no oculist would ever refuse to care for those unable to pay. The public needs to understand the subject. To many there is no difference between the oculist and the optician. It is high time that the public did know that the oculist is a physician.

---

Trachoma in Kentucky. John McMullen, U. S. P. H. S. (Report to the Surgeon General) investigated the trachoma conditions in seven counties of the mountains of Eastern Kentucky. He examined nearly four thousand people, and 12½ per cent. were found to be suffering from trachoma. Others had reported as high as 25 per cent. infected. Corneal complications are very frequent. To those who doubt the wisdom of classifying trachoma as a dangerous communicable disease it is suggested that they visit this locality and see what a great handicap it imposes upon its victims. It has been stated that 75 per cent. of untreated cases result in blindness. Living conditions are extremely unhygienic, and there are few opportunities for proper treatment. Some time ago Dr. Bailey found that in the Kentucky Institution for the Blind 45 per cent. of the inmates were blind of trachoma.

